# Protocol—the RAMESES II study: developing guidance and reporting standards for realist evaluation

**DOI:** 10.1136/bmjopen-2015-008567

**Published:** 2015-08-03

**Authors:** Trisha Greenhalgh, Geoff Wong, Justin Jagosh, Joanne Greenhalgh, Ana Manzano, Gill Westhorp, Ray Pawson

**Affiliations:** 1 Nuffield Department of Primary Care Health Sciences, University of Oxford, Oxford, UK; 2 Centre for Advancement in Realist Evaluation and Synthesis (CARES), University of Liverpool, Liverpool, UK; 3 Sociology and Social Policy, University of Leeds, Leeds, UK; 4 Community Matters Pty Ltd, Woodside, South Australia, Australia

**Keywords:** MEDICAL JOURNALISM, EDUCATION & TRAINING (see Medical Education & Training)

## Abstract

**Introduction:**

Realist evaluation is an increasingly popular methodology in health services research. For realist evaluations (RE) this project aims to: develop quality and reporting standards and training materials; build capacity for undertaking and critically evaluating them; produce resources and training materials for lay participants, and those seeking to involve them.

**Methods:**

To achieve our aims, we will: (1) Establish management and governance infrastructure; (2) Recruit an interdisciplinary Delphi panel of 35 participants with diverse relevant experience of RE; (3) Summarise current literature and expert opinion on best practice in RE; (4) Run an online Delphi panel to generate and refine items for quality and reporting standards; (5) Capture ‘real world’ experiences and challenges of RE—for example, by providing ongoing support to realist evaluations, hosting the RAMESES JISCmail list on realist research, and feeding problems and insights from these into the deliberations of the Delphi panel; (6) Produce quality and reporting standards; (7) Collate examples of the learning and training needs of researchers, students, reviewers and lay members in relation to RE; (8) Develop, deliver and evaluate training materials for RE and deliver training workshops; and (9) Develop and evaluate information and resources for patients and other lay participants in RE (eg, draft template information sheets and model consent forms) and; (10) Disseminate training materials and other resources.

Planned outputs: (1) Quality and reporting standards and training materials for RE. (2) Methodological support for RE. (3) Increase in capacity to support and evaluate RE. (4) Accessible, plain-English resources for patients and the public participating in RE.

**Discussion:**

The realist evaluation is a relatively new approach to evaluation and its overall place in the is not yet fully established. As with all primary research approaches, guidance on quality assurance and uniform reporting is an important step towards improving quality and consistency.

## Background

### Introduction

Many of the problems confronting researchers today are complex. For example, much health need results from the effects of smoking, suboptimal diets (including obesity), alcohol excess, inactivity or adverse family circumstances (eg, partner violence)—all of which in turn have multiple causes operating at both individual and societal level. Interventions or programmes designed to tackle such problems are themselves complex, having multiple, interconnected components delivered individually or targeted at communities or populations. Their success depends both on individuals’ responses and on the wider context in which people strive (or not) to live meaningful and healthy lives. What works in one family, or one organisation or one city may not work in another.

Similarly, the ‘wicked problems’ of contemporary health services research—how to improve quality and assure patient safety consistently across the service; how to meet rising need from a shrinking budget; and how to realise the potential of information and communication technologies (which often promise more than they deliver)—require complex delivery programmes with multiple, interlocked components that engage with the particularities of context. What works in hospital A may not work in hospital B.

Designing and evaluating complex interventions is challenging. Randomised trials that compare ‘intervention on’ with ‘intervention off’, and their secondary research equivalent, meta-analyses of such trials, may produce statistically accurate but unhelpful statements (eg, that the intervention works ‘on average’) which leave us none the wiser about where to target resources or how to maximise impact.

A relatively new approach (especially in health services research) to addressing these problems is realist evaluation. A form of theory-driven evaluation, based on realist philosophy,^1^ it aims to advance understanding of why these complex interventions work, how, for whom, in what context and to what extent—and also to explain the many situations in which a programme fails to achieve the anticipated benefit.

Realist evaluation assumes both that social systems and structures are ‘real’ (because they have real effects) and also that human actors respond differently to interventions in different circumstances. To understand how an intervention might generate different outcomes in different circumstances, realism introduces the concept of *mechanisms*—underlying changes in the reasoning and behaviour of participants that are triggered in particular contexts. For example, a school-based feeding programme may work by short-term hunger relief in young children in a low-income rural setting where famine has produced overt nutritional deficiencies, but for teenagers in a troubled inner-city community where many young people are disaffected, it may work chiefly by making pupils feel valued and nurtured.^2^

Realist evaluations have addressed numerous topics of central relevance in health services research, including what works for whom when ‘modernising’ health services,^3^ introducing breastfeeding support groups,^4^ using communities of practice to drive change,^5^ involving patients and the public in research,^6^ how robotic surgery impacts on team working and decision-making within the operating theatre^7^ and fines for delays in discharge from hospitals.^8^

### What is realist evaluation?

Realist evaluation was developed by Pawson and Tilley in the 1990s, to address the question “what works for whom in what circumstances and how?” in criminal justice interventions.^9^ This early work made the following points:

- Social programmes (closely akin to what health services researchers call complex interventions) are an attempt to address an existing social problem—that is, to create some level of social change.
- Programmes ‘work’ by enabling participants to make different choices (although choice-making is always constrained by such things as participants’ previous experiences, beliefs and attitudes, opportunities and access to resources).
- Making and sustaining different choices requires a change in a participant's reasoning (eg, in their values, beliefs, attitudes or the logic they apply to a particular situation) and/or the resources (eg, information, skills, material resources, support) they have available to them. This combination of ‘reasoning and resources’ is what enables the programme to ‘work’ and is known as a ‘mechanism’.
- Programmes ‘work’ in different ways for different people (ie, the contexts within programmes can trigger different change mechanisms for different participants).
- The contexts in which programmes operate make a difference to the outcomes they achieve. Programme contexts include features such as social, economic and political structures, organisational context, programme participants, programme staffing, geographical and historical context and so on.
- Some factors in the context may enable particular mechanisms to be triggered. Other aspects of the context may prevent particular mechanisms from being triggered. That is, there is always an interaction between context and mechanism, and that interaction is what creates the programme's impacts or outcomes: Context+Mechanism=Outcome.
- Since programmes work differently in different contexts and through different change mechanisms, programmes cannot simply be replicated from one context to another and automatically achieve the same outcomes. Theory-based understandings about ‘what works for whom, in what contexts, and how’ are, however, transferable.
- Therefore, one of the tasks of evaluation is to learn more about ‘what works for whom’, ‘in which contexts particular programmes do and don't work’ and ‘what mechanisms are triggered by what programmes in what contexts’.

A realist approach assumes that programmes are ‘theories incarnate’. That is, whenever a programme is implemented, it is testing a theory about what ‘might cause change’, even though that theory may not be explicit. One of the tasks of a realist evaluation is therefore to make the theories within a programme explicit, by developing clear hypotheses about how, and for whom, programmes might ‘work’. The implementation of the programme, and the evaluation of it, then tests those hypotheses. This means collecting data, not just about programme impacts or the processes of programme implementation, but about the specific aspects of programme context that might impact on programme outcomes and about the specific mechanisms that might be creating change.

Pawson and Tilley also argue that a realist approach has particular implications for the design of an evaluation and the roles of participants. For example, rather than comparing changes for participants who have undertaken a programme with a group of people who have not (as is performed in randomised controlled or quasi-experimental designs), a realist evaluation compares context-mechanism-outcome configurations within programmes. It may ask, for example, whether a programme works more or less well, and/or through different mechanisms, in different localities (and if so, how and why); or for different population groups (eg, men and women, or groups with differing socioeconomic status). Further, they argue that different stakeholders will have different information and understandings about how programmes are supposed to work and whether they in fact do so. Data collection processes (interviews, focus groups, questionnaires and so on) should be constructed partly to identify the particular information that those stakeholder groups will have, and thereby to refute or refine theories about how and for whom the programme ‘works’. The philosophical underpinnings of realist evaluation maybe found in box 1.

Box 1 **The philosophical underpinnings of realist evaluation.**

“Realism is a methodological orientation, or a broad logic of inquiry that is grounded in the philosophy of science and social science”.^10^

Philosophically speaking, realism can be thought of as sitting between positivism (‘there is a real external world which we can come to know directly through experiment and observation’) and constructivism (‘given that all we can know has been interpreted through human senses and the human brain, we cannot know for sure what the nature of reality is’). Realism holds that there is a real social world but that our knowledge of it is amassed and interpreted (sometimes partially and/or imperfectly) via our senses and brains, filtered through our language, culture and past experience.

In other words, realism sees the human agent as suspended in a wider social reality, encountering experiences, opportunities and resources and interpreting and responding to the social world within particular personal, social, historical and cultural frames. For this reason, different people in different social, cultural and organisational settings respond differently to the same experiences, opportunities and resources. Hence, a programme (or, in the language of health services research, a complex intervention) aimed at improving health outcomes is likely to have different levels of success with different participants in different contexts—and even in the same context at different times.

### The need for standards and training materials in realist evaluation

The RAMESES JISCmail listserv (http://www.jiscmail.ac.uk/RAMESES—an email list for discussing realist approaches) postings suggest that enthusiasm for realist evaluation and belief in its potential for application in many fields have outstripped the development and application of robust quality standards in the field. Two recent publications have systematically shown that many so-called ‘realist evaluations’ were not applying the concepts appropriately and were (as a result) producing misleading findings and recommendations.^11^ ^12^

Pawson and Manzano-Santaella^12^ in their paper ‘A realist diagnostic workshop’ used case examples of flawed realist evaluations to highlight three common errors in such studies. First, while it is possible to show associations and correlations in data from many types of evaluation, the focus of a realist evaluation is to explore and explain why such associations occur. Second, they explain what may constitute valid data for use in realist evaluation. Producing a realist explanation requires a mix of data types, not only qualitative data, to provide explanations and support for the relationships within and between context mechanisms outcome configurations. Third, realist explanations require context-mechanism-outcome configurations to be produced. They note that some realist evaluations have become bogged down in finely detailed lists of contexts, mechanisms and outcomes but failed to produce a coherent explanation of how these Cs Ms and Os were linked and related (or not) to each other. Pawson and Manzano-Santaella call for greater emphasis on elucidating programme theory (the theory about what a programme or intervention is expected to do and in some cases, how it is expected to work) expressed as CMO configurations.

Marchal *et al*^11^ undertook a review of the realist evaluation literature to quantify and analyse the field. They identified 18 realist evaluations and noted a range of challenges that arose for researchers. Absence of prior theoretical and methodological guidance appeared to have led to recurring problems in the realist evaluations they appraised. First, ‘The philosophical principles that underlie realist evaluation are variably interpreted and applied to different degrees. Most authors only fleetingly refer to the philosophical foundation of realist evaluation, which arguably is among its most distinctive features and provides much of its explanatory power’. In addition, they noted that different researchers had conceptualised concepts used in realist evaluation, such as ‘middle-range theory’, ‘mechanism’ and ‘context’ differently. This, they concluded, was often related to fundamental misunderstandings. Where misunderstandings occurred, rigour of the realist evaluation undertaken often suffered.

These two papers show that realist evaluation is often an intellectually challenging task. Both sets of authors point out that more guidance is needed to allay misunderstandings about the purpose, underlying philosophical assumptions and analytic concepts and processes of realist evaluation.

## Methods/design

### Study design

**Figure 1:**
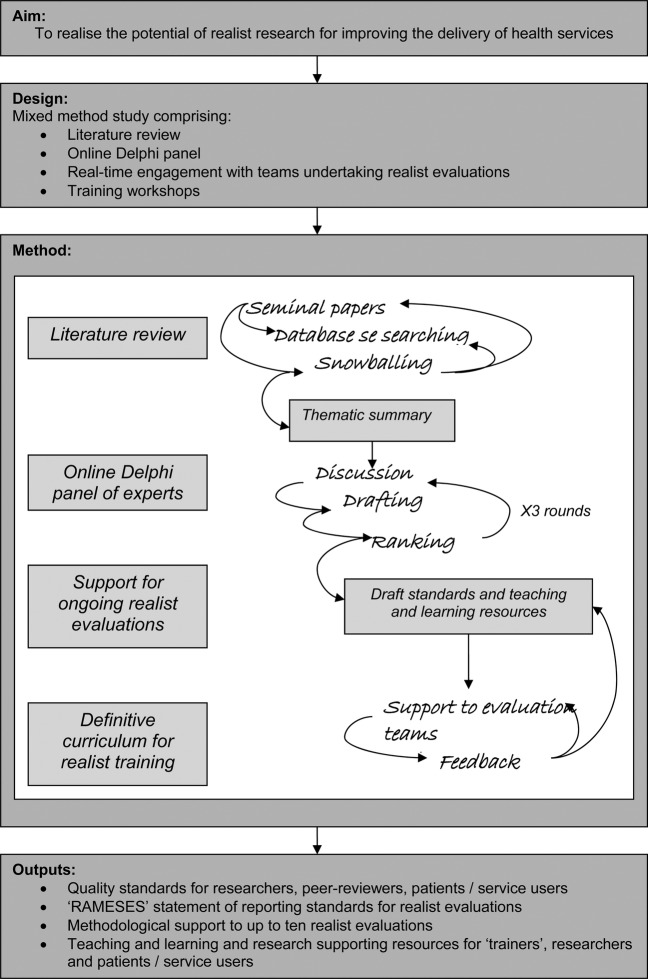
Study protocol.

Mixed-methods study comprising literature review, online Delphi panel, real-time engagement with teams undertaking realist evaluations and training workshops (figure 1).

### The online Delphi method

To develop our quality and reporting standards we will use the online Delphi method. We had previously successfully used this method to develop quality and reporting standards and training materials for meta-narrative reviews and realist syntheses in the RAMESES I (Realist And Meta-narrative Evidence Syntheses: Evolving Standards) project.^13^

In brief, the essence of the Delphi technique is to engender reflection and discussion among a panel of experts with a view to getting as close as possible to consensus. Both the agreements reached and the nature and extent of residual disagreement are documented.^14^ It was used, for example, to set the original care standards which formed the basis of the Quality and Outcomes Framework for UK general practitioners.^15^ Our experience and the evidence indicate that the online medium is more likely to improve than jeopardise the quality of the development process. Delphi panels conducted at a distance have been shown to be as reliable as face-to-face panels^16^ and offer advantages, such as less cost, speed and greater flexibility for those involved.^17^ Our experiences of using the online Delphi method chimes with that of others and indicate that it is the underlying design and rigour of the Delphi process which is key to quality and not the medium through which it happens.^14^ ^18^

### Study aims

This project sets out to:

- Develop quality standards, reporting guidance and training materials for realist evaluation
- Build capacity for undertaking and critically evaluating realist evaluation in the healthcare context
- Produce resources and training materials for lay participants, and those seeking to involve them, in realist evaluations.

The project has 10 operational objectives which are described in detail below. The project's 10 operational objectives will be delivered in three workstreams, underpinned by a management and governance infrastructure. The detail is set out below.

*Objective 1:* Establish a management and governance infrastructure, including a project advisory group with lay representation and a patient/service user panel.

A core working group will meet fortnightly, and the advisory group (with lay representation) and a separate patient/service user panel will each meet 6 monthly. This infrastructure will advise and support (but not replace) regular meetings among the researchers, as needed, to execute the study, conduct the data analysis, discuss emerging findings and prepare outputs.

The project advisory group will have wide cross-sector representation (including experts in realist evaluation, research support, NHS professionals and representatives from the patient panel). It will monitor progress against milestones and spend against budget, provide advice, promote the project, communicate with stakeholders and help maximise dissemination and impact of findings. In addition, where needed it will act as a sounding board and ‘critical friend’ to the project team.

The patient panel will provide advice and feedback to the working group to on how to present the study and findings in a way that is maximally accessible to lay people. Representatives from it will attend the project advisory group (with training and support if required). Where necessary we will provide induction and training to the group members and ensure that they are made aware that their participation is entirely voluntary and may withdraw at any time.

#### Workstream 1 (Objectives 2, 3 and 4)

*Objective 2:* Recruit an interdisciplinary Delphi panel

For the online Delphi panel, we will apply the same successful approach as we did for the RAMESES study.^13^ We will recruit 35 panellists the groups listed in the objective above (including patient organisations). Recruitment will be carried out by the core working group, drawing on our knowledge of the field, our different professional networks, the RAMESES JISCmail listserv and our links to user organisations. Input from a wide range of experts in relevant fields will be sought, consisting of researchers, people who support and help design research studies, publishers, peer reviewers, policymakers, patient advocates and practitioners with (various types of) experience relevant to realist evaluation. Those who meet one or more criteria for expertise will be briefed on the project, what is expected from them and informed that participation is voluntary and unpaid and that they may withdraw at any time. We will ensure representation from all relevant stakeholder groups, if necessary by asking existing panel members to nominate and invite others.

*Objective 3:* Summarise the current literature and expert opinion on best practice in realist evaluation, to serve as a baseline/briefing document for the panel.

With expert librarian help, we will identify reviews, scholarly commentaries, models of good practice and examples of (alleged) misapplication of realist evaluation.^11^ ^12^ To identify the relevant documents we will refine and develop the search used by Marchal *et al*^11^ for a previous review on a similar topic, and also apply contemporary search methods designed to identify ‘richness’ when exploring complex interventions.^19^ ^20^ We will thematically summarise (1) what is considered by experts to be current best practice (and the range and diversity of such practice); (2) what experts and other researchers believe count as high quality and needs to be reported; and (3) what issues researchers struggle with (based on thematic analysis of postings on the RAMESES JISCmail list archive as well as the published literature). The purpose of this step is not to produce definitive answers to these questions but to prepare a baseline set of briefing materials for the Delphi panel, who will deliberate on them and add to them in the next step.

*Objective 4:* Run three (and more if needed) rounds of the online Delphi panel to generate and refine items for a set of quality and reporting standards.

The Delphi panel will be run online using SurveyMonkey (Survey Monkey, Palo Alto, California, USA). Participants in round 1 will be provided with briefing materials and invited to suggest what might be included in the reporting standards. Responses will be analysed and fed into the design of questionnaire items for round 2.

In round 2 of the Delphi Panel participants will be asked to rank each potential item twice on a 7-point Likert scale (1=strongly disagree to 7=strongly agree), once for relevance (ie, should an item on this theme/topic be included at all in the guidance?) and once for validity (ie, to what extent do you agree with this item as currently worded?). Those who agreed that an item was relevant, but disagreed on its wording, will be invited to suggest changes to the wording via a free-text comments box. In this second round, participants will again be invited to suggest additional topic areas and items.

Each participant's responses will be collated and the numerical rankings entered onto an Excel spreadsheet. The response rate, average, mode, median and IQR for each participant's response to each item will be calculated. Items that score low on relevance will be omitted from subsequent rounds. We will invite further online discussion on items that score high on relevance but low on validity (indicating that a rephrased version of the item may be needed) and on those where there was wide disagreement about relevance or validity. The panel members’ free text comments will also be collated and analysed thematically.

Following analysis and discussion within the project team we will then draw up a second list of statements and will be circulated for ranking (round 3). Round 3 will only contain items where consensus has not yet been reached. We plan that the process of collation of responses, further e-mail discussion and re-ranking will be repeated until a maximum consensus is reached (round 4 et seq.). In practice, very few Delphi panels, online or face to face, go beyond three rounds as participants tend to ‘agree to differ’ rather than move towards further consensus. We will use email reminders to optimise our response rate from Delphi panel members. We will consider consensus to have been achieved when the median score is 6 or above.

We plan to report residual non-consensus as such and the nature of the dissent described. Making such dissent explicit tends to expose inherent ambiguities (which may be philosophical or practical) and acknowledges that not everything can be resolved; such findings may be more use to those who use realist evaluation than a firm statement that implies that all tensions have been fixed.

#### Workstream 2 (Objectives 5 and 6)

*Objective 5:* In parallel with the Delphi panel:

- Provide ongoing advice and consultancy to up to 10 realist evaluations, thereby capturing the ‘real world’ problems and challenges of this methodology.
- Host the RAMESES JISCmail list on realist research, capturing relevant discussions about theoretical, methodological and practical issues.
- Feed problems and insights from 5A and 5B into the deliberations of the Delphi panel and the design of training resources and courses.

We will provide advice and or methodological support to up to 10 realist evaluations. To sample 10 that unfold in parallel with our Delphi exercise, we will (1) ask NIHR to link us with planned evaluations funded by them that align with our own timeline; (2) ask on the RAMESES list; (3) capture unsolicited requests for help (of which we receive many). We will aim for maximum variety in experience of research teams, topics, settings and approach to patient and public involvement. We will work flexibly with teams, mostly by phone, Skype and email, to support them with methodological advice and troubleshooting. We will systematically capture the questions and issues from these 10 primary studies and feed them into the deliberations of the Delphi panel (where timings permit) and, if relevant, the training materials and courses described below.

We provided a comparable service to realist review teams in the RAMESES I study, and plan to follow a similar approach.^13^ In RAMESES I, there was considerable variation in the level of expertise and confidence in the research teams. Some were highly skilled and used our input mainly as ‘sounding board’ for their own developing ideas and methodology. Others lacked basic understanding of realist concepts and methods; they were offered face-to-face training workshops and bespoke support with data analysis and interpretation. We captured numerous methodological issues that fed into the design of training materials and also informed some methodological papers by our team and the teams we worked with (some of whom have now joined this new collaborative bid).^21^ ^22^ We will aim for a similar set of outputs in this work package in RAMESES II.

*Objective 6:* Write up the quality standards and reporting guidelines for an open-access journal.

We will follow the method applied successfully in RAMESES I to produce an account of the background, methods, main findings and conclusions of the Delphi project, including publishing a detailed protocol in an open access journal^23^ ^24^ and engaging the editors of specialist journals in potential parallel publication to reach an extended range of readers.^25^ ^26^ We will also, as in RAMESES I, enter into dialogue with the EQUATOR network (http://www.equator-network.org), a clearinghouse for reporting standards which is used as a first port of call by researchers seeking such standards, and which already lists the RAMESES standards for secondary research.

Achieving consensus on both quality standards and reporting guidelines may be more difficult for realist evaluation than it was for realist review, since the former covers a huge variety of settings, topics, approaches and configurations.^1^ Hence it is possible that, unlike in RAMESES I, consensus among Delphi panel members may not be achieved for all items. This is not inherently a problem: in a previous Delphi study to develop standards for undertaking and reporting narrative research, we simply reported, and commented on, the areas of residual disagreement between panel members, which were explained by their different disciplinary and/or sectoral backgrounds.^27^

#### Workstream 3 (Objectives 7–10)

*Objective 7:* Collate examples of learning/training needs for researchers, postgraduate students, reviewers and lay members in relation to realist evaluation.

We will seek examples of the kinds of requests that are made by researchers for support on realist evaluation. We already have a rich archive of postings on the RAMESES JISCmail listserv from both novice and highly experienced researchers, going back 3 years. We will also proactively ask the list members for additional examples; use our empirical data from workstream 2 on the real-world struggles of realist researchers (see Objective 5 above); and draw on our literature review (Objective 3) and Delphi panel discussions (Objective 4), to identify relevant examples. Finally, we will seek input from UK Research Design Service (RDS) staff, particularly with those who respond to an invitation sent out by the RDS Steering Group on our behalf. We will ask such RDS staff (some of whom are already members of the RAMESES list) to describe the kind of problems people bring to them, and where they feel that further guidance, support and resources are needed.

We will use a thematic approach to classify examples into a coherent taxonomy of problems and issues, each with a corresponding training need(s). This will be developed iteratively in regular meetings of the research team. At least two researchers will independently classify examples within this taxonomy and through subsequent discussion with the wider team, both the taxonomy and the classification of examples within it will be refined. The goal of this step will be to feed into a coherent and comprehensive curriculum for training realist researchers and for ‘training the trainers’.

*Objective 8:* Develop, deliver and evaluate training materials for realist evaluation. Deliver 3×2-day ‘realist evaluation’ workshops AND 3×2-day ‘training the trainers’ workshops for a range of audiences (including interested NIHR Research Design Service staff)

To develop training materials, we will analyse and take forward various problems, issues and learning needs raised in the examples identified in Objective 7. Some will be philosophical or theoretical, some methodological, some practical, some ethical and so on. Different kinds of learning need require different materials and resources and delivered by different media (face-to-face, internet) and in different learning arrangements (self-study, online drill-and-practice, interactive group tasks and so on). Developing the resources will involve setting specific learning objectives, preparing study notes (eg, explanations, diagrams) and developing and piloting exercises to engage learners. For each main challenge, we will produce a menu of materials oriented to different audiences and learning styles. Several of the applicants on this bid are experienced trainers and consultants on realist evaluation; we will draw on, and refine, the existing training materials that we have developed and acquired over the years.

It is important to stress that realist evaluation cannot be achieved simply by following a protocol in a technically correct manner. Rather, becoming competent at realist evaluation involves acquiring the ability to think, reflect and interpret data in a way that is resonant with realist philosophy and principles. For this reason, much of the workshops will take the form of ‘show and tell’, facilitated discussion and ‘apprenticeship’ to experienced and skilled realist researchers.

We will run 3×2-day ‘how to do a realist evaluation’ workshops for a main audience of researchers and evaluators, and including research users—both lay and professional and 3×2-day ‘training the trainers in realist research’ workshops for a main audience of those who train and support such work. In both sets of workshops, diversity of background will be used productively in group-based case discussions and other hands-on, interactive formats.

The training the trainers workshops in particular will be open to RDS staff who seek to become confident in supporting realist studies; they will also seek interdisciplinary participation from researchers, practitioners, policymakers and patient advocates. The detailed curriculum for the workshops will emerge from our empirical work, but the training the trainers programme will include all the steps needed to set up and run a responsive service to support and evaluate realist reviews and evaluations, including costing different components of support.

*Objective 9:* Develop, deliver and evaluate information and resources for patients and other lay participants in realist evaluation. In particular, draft template information sheets and consent forms that could be adapted for ethics and governance activity, and deliver up to six workshops for PPI organisations.

We will engage with our patient/service user panel to help us develop resources that are relevant, understandable and useful to this group. Examples are: the quality and reporting standards; some of the training resources, especially lay summaries of what a realist evaluation is; template information sheets and consent forms for participants in realist evaluations.

As well as developing ‘generic’ patient/lay resources, we will offer up to six half-day workshops on realist evaluation for patient organisations. We will work with each organisation to develop a curriculum and format. Organisations for these workshops will not be formally sampled as we have found in the past that we receive ‘ad hoc’ requests for such input, which we often have to turn down because of lack of protected time. Hence this will be a responsive component of the study, dependent on which organisations approach us. Those who do so will probably hear about us from the following sources: (1) the RAMESES listserv, whose membership includes a number of patient/lay advocates; (2) our patient panel and their personal networks; (3) social media invitations (eg, TG has an active presence on Twitter and more than 10 000 followers, many of whom represent patient organisations); and (4) newsletters and email feeds from organisations such as INVOLVE (http://www.invo.org.uk).

*Objective 10:* Disseminate training materials and other resources—for example, via public access websites.

We will replicate the dissemination approach we used for the RAMESES I study, namely: (1) publish the standards in a peer-reviewed journal (in parallel if possible); (2) develop the existing RAMESES project website to host and facilitate open access to all resources; (3) continue to run the RAMESES JISCmail list (on which we posted the links to the above); and (4) submit the reporting standards to the EQUATOR NETWORK (an international clearinghouse for peer-reviewed reporting standards, http://www.equator-network.org).

In addition, we will emphasise the development, piloting and publishing of lay summaries of the key publications. Depending on the journal, it may be possible to publish these lay summaries alongside the academic papers (eg, New England Journal of Medicine offers such an option). We will make lay summaries available on the RAMESES project website, and will negotiate with COREC (research ethics) and INVOLVE to publish templates of information sheets and consent forms for patient participants in realist evaluation. We will ask the Research Design Service to link to resources relevant to their staff and clients (and have agreement from the RDS to do this in principle).

## Discussion

Realist evaluation is a relatively new approach to evaluation, especially in health services research. It potentially offers great promise in unpacking the ‘black box’ of the many complex interventions that are increasingly being used to improve health and patient outcomes. As relatively experienced users of this approach, we have noted a number of common and recurrent challenges that face grant awarding bodies, peer-reviewers, reviewers and users. These centre on two closely related questions, namely how to judge if a realist evaluation or a proposal for such an evaluation, is of ‘high quality’ (including, for completed evaluations, how ‘credible’ and ‘robust’ findings are) and how to undertake such evaluations. Our experience to date suggests that we can go a long way towards answering these questions by giving due consideration to the theoretical and conceptual underpinnings of realist evaluation, outlined briefly below.

Realist evaluation is based on a realist philosophy of science, which permeates and informs its underlying epistemological assumptions, methodology and quality considerations. One of the most common misapplications we have noted is that evaluators have not always appreciated the underlying philosophical basis of this approach (and the implications of these for how the evaluation should be conducted). Instead, they have based their evaluations explicitly or implicitly on fundamentally different philosophical assumptions—most commonly the positivist notion that interventions in and of themselves cause outcomes.

Even when a realist philosophy of science has been adhered to in a realist evaluation, reviewers—ourselves included—often struggle with recurring conceptual and methodological issues. ‘Mechanisms’ present a particular challenge in realist evaluation—how to define them, where to locate them, how to identify them and how to test and refine them.^28^ Realist evaluation trades on the use of theoretical explanations to make sense of the observed data. Realist evaluators commonly grapple with how to define a theory (what, eg, is the difference between a ‘programme theory’ and a ‘middle-range theory’?) and what level of abstraction is appropriate in what circumstances. On a more pragmatic level, those who seek to undertake realist evaluations wrestle with a broad range of ‘how to’ issues: how to produce a programme theory; what type of data needs to be collected; how to use collected data to refine a programme theory; how and to what extent to refine the scope as the evaluation as it unfolds; what changes can legitimately be made to data collected methods; how to organise, analyse and synthesise the collected data; how to make recommendations that are academically defensible and useful to policymakers and the research community; and so on.

As we have mentioned above, realist evaluation is a relatively new approach and so we are aware that methodological development is very likely to occur. Realist evaluation as an approach has also been used in a wide range of disciplines—both in and outside of health. These two issues will have a significant impact on the RAMESES II project and have already been debated and discussed since the start of the project. We want to ensure that the project's outputs do not stifle innovation and methodological development in realist evaluation. To address this issue we can draw on our experience in developing similar resources in the first RAMESES project.^13^ For example, in the quality and publication standards we produced for the first RAMESES project, we deliberately stated that researchers were able to make any changes they felt were needed to a review's processes, but should explain what were the changes, where and why they had been made. To address the issue of the wide range of disciplines that use realist evaluation, we will draw on realist and/or evaluation expertise from a broad range of disciplines for our Delphi panel. This will ensure that there is not just one dominant ‘voice’ (eg, from health researchers) on the panel, thus enabling any of the project's outputs to be suitable for use in a wide range of circumstances.

## Conclusion

While realist evaluation holds much promise for developing theory and informing policy in many fields of research, misunderstandings and misapplications of this approach is common. The time is ripe to start on the iterative journey of producing guidance on quality and reporting standards as well as developing quality-assured learning resources to ensure that funding decisions, execution, reporting and use of this evaluation approach is optimised. Acknowledging that research is never static, the RAMESES II project does not seek to produce the last word on this topic but to capture current expertise and establish an agreed ‘state of the science’ on which future researchers will no doubt build.

We anticipate that the Delphi panel will start in September 2015 (at the latest) and that a paper describing the guidance will be submitted by April 2016. The online discussion forum is open to anyone with an interest in realist evaluation and may be found at http://www.jiscmail.ac.uk/RAMESES.
